# A convolutional neural network provides a generalizable model of natural sound coding by neural populations in auditory cortex

**DOI:** 10.1371/journal.pcbi.1011110

**Published:** 2023-05-05

**Authors:** Jacob R. Pennington, Stephen V. David

**Affiliations:** 1 Washington State University, Vancouver, Washington, United States of America; 2 Oregon Hearing Research Center, Oregon Health and Science University, Oregon, United States of America; University of California at Berkeley, UNITED STATES

## Abstract

Convolutional neural networks (CNNs) can provide powerful and flexible models of neural sensory processing. However, the utility of CNNs in studying the auditory system has been limited by their requirement for large datasets and the complex response properties of single auditory neurons. To address these limitations, we developed a population encoding model: a CNN that simultaneously predicts activity of several hundred neurons recorded during presentation of a large set of natural sounds. This approach defines a shared spectro-temporal space and pools statistical power across neurons. Population models of varying architecture performed consistently and substantially better than traditional linear-nonlinear models on data from primary and non-primary auditory cortex. Moreover, population models were highly generalizable. The output layer of a model pre-trained on one population of neurons could be fit to data from novel single units, achieving performance equivalent to that of neurons in the original fit data. This ability to generalize suggests that population encoding models capture a complete representational space across neurons in an auditory cortical field.

## Author summary

Sounds in the natural world are composed of complex, dynamic spectro-temporal features. The brain’s auditory system is able to identify and extract meaningful patterns from acoustic inputs, including in the presence of noise and other competing sounds. A better understanding of auditory neural computation may inform algorithms for speech processing and auditory prosthetics. Despite their importance, current computational models have limited success explaining neural sound coding, particularly in auditory cortex. This study used convolutional neural networks (CNNs) to model the functional relationship between a large set of natural sounds and the activity of neurons in auditory cortex. The CNNs substantially outperformed several previously proposed models of auditory coding. Moreover, they were able to generalize. After training on data from one set of neurons, they could be trained easily on a new set of neurons. This finding suggests that the CNN models characterize a general space of spectro-temporal patterns encoded by the population of neurons in auditory cortex. A better understanding of this brain-derived space may be useful for improved signal processing algorithms.

## Introduction

A complete understanding of neural sensory processing requires computational models that can account for brain activity evoked by arbitrary natural stimuli [1]. In the auditory cortex, encoding models such as the widely used linear-nonlinear spectro-temporal receptive field (LN model) can account for sound-evoked spiking activity in some neurons, but often fail to predict time-varying responses to complex stimuli such as natural sounds [2,3]. Encoding models are used to study auditory coding by many neurophysiological signals beyond single neuron spikes, including calcium imaging [4], spiking ensembles [5], human LFP [6,7], MEG [8], and fMRI BOLD [9,10]. Thus, improved encoding models are of broad value to research on the auditory system.

Variants of the LN model have been proposed that provide a more accurate characterization of auditory coding. Some of these variants build on traditional systems identification methods, accounting for second- and higher order nonlinearities [11–13]. Others incorporate nonlinear elements derived directly from biological circuits, like short-term synaptic plasticity and gain control by local inhibitory populations [3,14,15]. Finally, another approach has been to combine linear-nonlinear units in multi-filter LN models [16–20] or artificial neural networks [7,21]. This work has shown that auditory neurons encode information in a sensory subspace and that a single linear filter is not adequate for capturing the diversity of inputs that modulate the neural output. While theoretically appealing, multi-filter and neural network models can be challenging to fit, especially when data set size is limited [13]. The diverse and sometimes highly optimized methods required to fit these more sophisticated models make comparisons between models difficult, as differences between them could reflect either their distinct architectures or the distinct methods used for fitting. Thus, direct comparisons between all these different models remain limited, and new models are typically compared only with the LN model as a baseline (but see [7,21,22]).

In the current study, we explored convolutional neural networks (CNNs) as a method for improving upon existing encoding models of neural spiking data. Advances in machine learning, in particular the development of backpropagation algorithms for CNNs, have opened up the possibility of applying neural network analysis to neurophysiological data [23,24]. CNNs have been adopted widely for signal processing problems, including speech recognition and other acoustic analysis [25,26]. In the auditory system, a small number of studies have indicated that CNNs can describe human BOLD fMRI data [27,28] and ECoG data [7]. CNNs have been used more extensively in the visual system to model natural image representation in retinal ganglion cells [29,30] and visual cortex [31–34]. It remains an open question whether CNNs can provide a useful characterization of single-neuron activity in auditory cortex.

One particular appeal of CNNs is that they can serve as “foundation models,” pre-trained on one task but then transferred to a wide range of new problems [35]. Such approaches are widely used for machine learning problems, including auditory [36] and visual signal processing [37]. Pre-trained CNNs have also been useful for analyzing neural data when limited data set size prevents fitting large models directly [27,31]. Neural networks trained on responses to natural videos and images have also demonstrated generalizability to neural data collected from different animals [38,39]. Motivated by the success of these approaches, we argue that an effective CNN-based encoding model should be fully generalizable. That is, a CNN that completely describes neural sensory processing should account for the encoding properties of neurons that were not included in the original model fit.

In order to fit CNN models and evaluate their generalizability, we recorded the time-varying spiking activity of a population of single neurons in auditory cortex during presentation of a large natural sound library. To leverage statistical power for model fitting, we developed a population encoding model, in which the activity of many neurons is predicted by a single CNN with input layers shared across neurons. Using this approach, we compared several CNN architectures, based on convolutional models widely used for visual processing [7] and based on LN models used in the auditory system [21]. We found that population CNN models all performed similarly and that they accounted for cortical activity substantially better than traditional LN models and multi-filter models [19,20]. In addition, pre-trained population models successfully generalized to novel neural data. Thus, CNN models fit with large neural populations and diverse stimuli can provide a generalizable model of sound encoding by auditory cortex.

## Results

To characterize the neural encoding of natural sounds, we recorded spiking activity from auditory cortex of awake, passively listening ferrets during presentation of a diverse set of natural sound samples. Neural data was collected with 64- or 128 channel linear silicon arrays that recorded simultaneous activity of 10–95 neurons across multiple cortical laminae during each experiment. Recordings were performed in primary auditory cortex (A1) and a secondary auditory field (PEG) located anterior-ventral to A1 [40,41]. The same stimuli were presented in all recordings (A1: 22 recording sites, 849 units; PEG: 11 sites, 398 units; 5 animals). The input stimulus spectrogram and output time-varying spike rate were sampled at 100 Hz.

### Convolutional neural networks with a shared tuning space for neural populations

**Fig 1:**
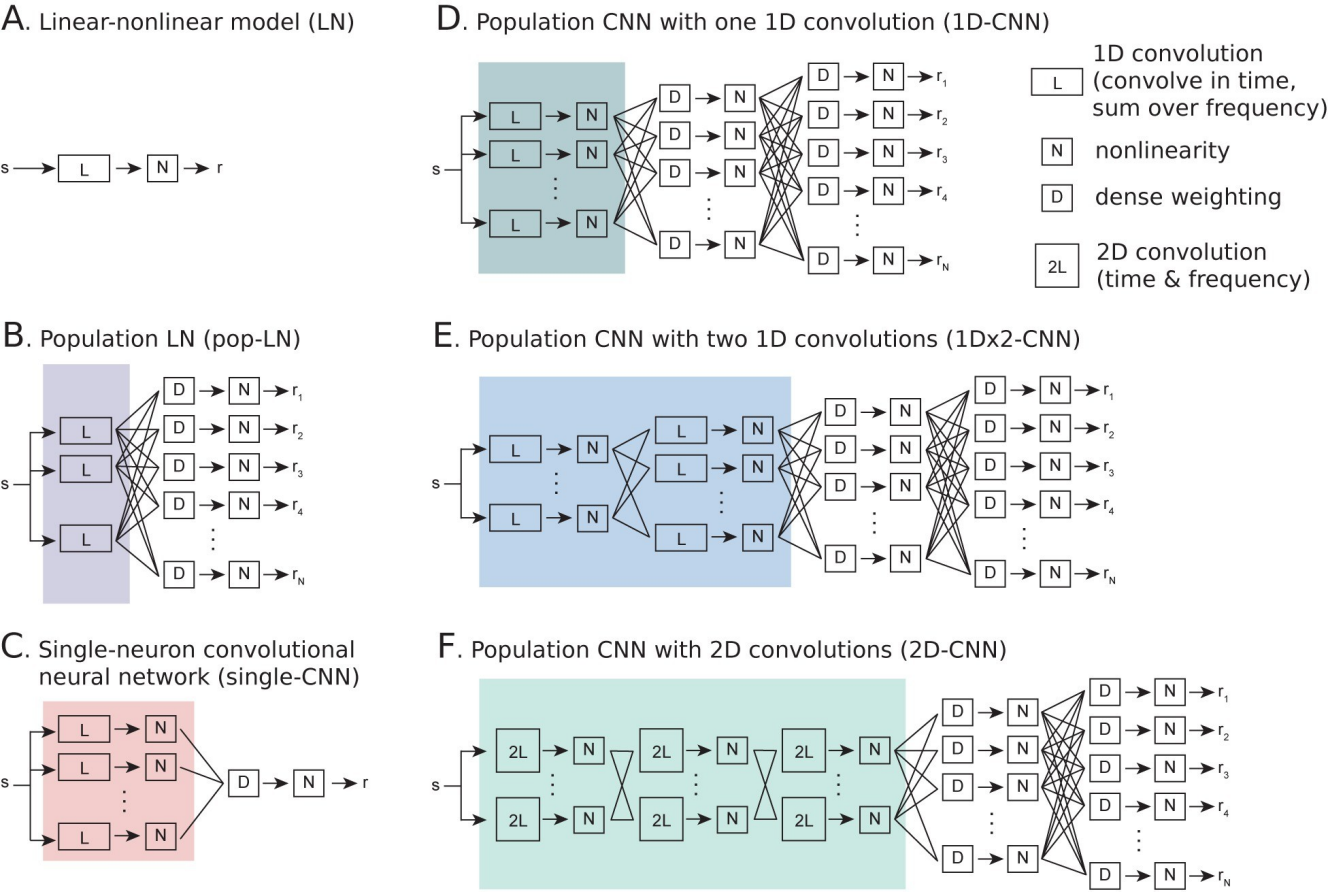
Convolutional neural networks (CNNs) provide a natural extension of standard linear-nonlinear (LN) models of auditory encoding. **A.** LN model consists of a single convolutional filter (L) followed by a static nonlinearity (N). This convolution is one-dimensional: a separate filter is convolved in time for each frequency channel, then the results are summed across frequency. Arrows indicate passthrough between units within a layer. **B.** Population LN (pop-LN) model is composed of a bank of temporal convolutional units (purple shading) followed by one dense unit (D) and static nonlinearity per neuron, where dense refers to a linear weighting of the outputs of the previous layer. **C.** Single-neuron convolutional neural network (single-CNN), or LNLN cascade [24], consists of a bank of LN units (red shading) linearly combined in a subsequent dense unit and followed by another static nonlinearity. **D-F.** Population CNN models consist of one or more convolutional layers and two dense, fully connected layers. Dense units in the final layer generate the output for each neuron. Convolutional units are either one-dimensional (“1D”, convolved in time and summed over frequency, derived from standard spectro-temporal models for auditory processing) or two-dimensional (“2D”, convolved in both time and frequency, derived from standard CNN models for visual processing). 1D models have either one convolutional layer (1D-CNN, **D,** dark green shading) or two (1Dx2-CNN, **E,** blue shading), while the 2D-CNN model includes three convolutional layers in sequence (**F**, light green shading).

We used a convolutional neural network (CNN) to describe the functional relationship between the natural sound spectrogram and the time-varying spiking activity (Fig 1). Machine learning models have proven effective for studying a wide range of analytically similar problems. However, fitting these models requires large datasets, and the amount of data available from many neurophysiological studies is limited. This limitation is compounded by the fact that sensory-evoked neural activity is not reliable, varying substantially across repeated stimulus presentations. The current study took advantage of the fact that identical stimuli were presented during multiple experiments to fit CNNs that simultaneously modeled an entire population of neurons. In this framework, the stimulus spectrogram provides input to a series of convolutional and dense (*i*.*e*., fully connected) layers that are shared across all neurons. A subsequent dense layer weights the output of the final shared layer to predict the activity of each neuron individually (Fig 1D–1F). Thus, the earlier layers comprise a shared, general model of sound processing in auditory cortex that is mapped to individual responses only in the final layer. This design is similar to the “core-readout” network developed to model encoding of natural visual stimuli, but is purely feedforward [38].

We considered three architectures of population CNN (Fig 1D–1F). Models adapted from LN and multi-filters models widely used in the auditory system employed “1D” filters that were convolved in time and summed along the spectral axis (Fig 1B–1E). The 1D shorthand refers to the fact that filters are convolved only in time, but these filters do each also integrate over input channels by weighted summation. This design is motivated by reduced-rank LN models which have proven to be efficient formulations of the LN architecture [42,43]. We evaluated two 1D CNN architectures: one with a single convolutional layer (1D-CNN, Fig 1D) and one with two convolutional layers in sequence (1Dx2-CNN, Fig 1E). In addition, an architecture adapted from standard CNN models for visual processing employed “2D” filters that were convolved along the temporal and spectral dimensions of the input spectrogram (Fig 1F). CNNs used in visual processing problems typically apply multiple layers of small, two-dimensional (2D) convolutional kernels to an input image [31]. This model can be transferred directly to the auditory system. In this case, small 2D filters are convolved along the time and frequency dimensions of the sound spectrogram (2D-CNN, Fig 1F) [7,27].

We compared performance of the population CNN models to a reduced-rank LN model, which we previously showed to be an optimal formulation of the LN architecture (LN, Fig 1B, [43]). We also implemented two intermediate architectures to control for differences between population CNN models and the standard LN model. To control for increased statistical power gained by pooling data across neurons in population models, we fit a population LN model (pop-LN, Fig 1B), in which the simultaneous activity of the recorded neural population is modeled as the linear weighted sum of a shared bank of 1D convolutional filters followed by nonlinear rectification. To distinguish possible benefits of the population model approach from benefits of the neural network architecture, we fit separate CNN models for each neuron in the dataset (single-CNN, Fig 1C). This type of model is often referred to as a LNLN cascade [24], and is an instantiation of a multi-filter model [16,19,20]. To accommodate sampling limitations of single unit data, these models contained substantially fewer units, and thus fewer total free parameters, than the larger population models. Finally, to compare CNNs to an existing alternative to the LN model, we fit a short-term plasticity (STP) model, in which nonlinear adaptation mimicking synaptic depression or facilitation was applied to each input channel of a reduced rank LN model [3].

All models were fit using standard back-propagation methods [44], which minimized the mean-squared error between predicted and actual time-varying neural activity. Fitting was carried out in two stages. First, parameters for the entire model were fit for all neurons simultaneously. Second, weights in the final layer were re-fit for each neuron individually (see Methods). Fitted models were then used to predict the response evoked by stimuli in a validation dataset that was not used for fitting. Model performance was evaluated on this separate dataset by measuring prediction correlation, the noise-corrected Pearson correlation coefficient between predicted and actual time-varying response, for each neuron [43,45]. A prediction correlation of 1.0 indicates that a neuron’s activity was predicted as accurately as possible given the uncertainty in the actual response, and a value of 0 indicates chance performance.

**Fig 2:**
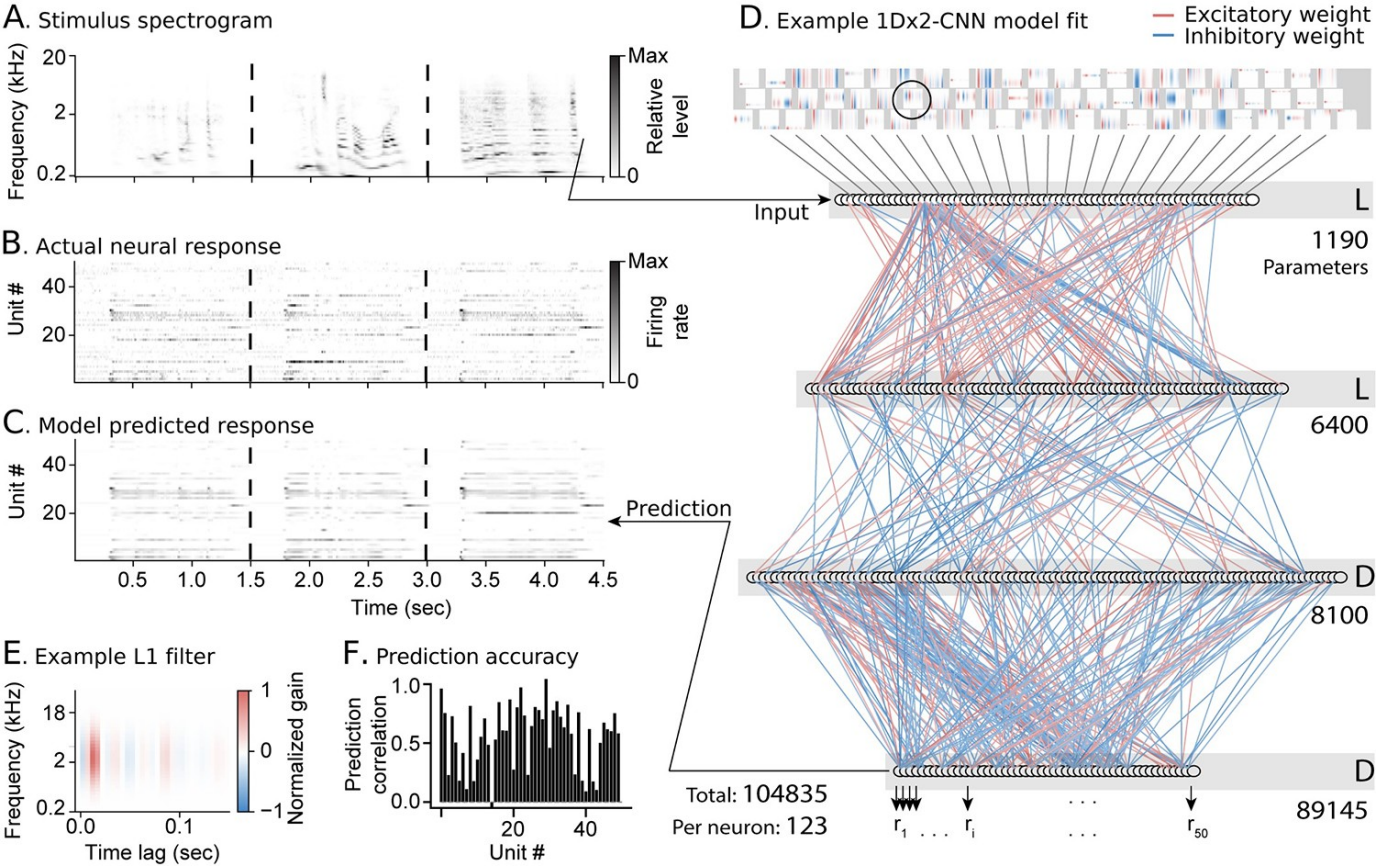
Example population CNN model fit for 849 A1 neurons. **A**. Example spectrogram of three natural sounds in the validation set used to measure model performance. **B**. Heat map shows the actual time-varying spike rate of one neuron per row, for a sample of n = 50 / 849 neurons from the A1 dataset. Spike rate is normalized for each neuron between 0 (white) and maximum (black). **C**. Heat map of predicted activity for the same units, plotted as in B. **D.** Schematic of model layers with fitted weights. Convolutional filters in the first layer (top) establish a shared set of spectro-temporal channels. These filters are generated from the outer product of the temporal convolution and spectral summation comprising each 1D convolutional unit. Line color connecting subsequent layers indicates the weight, with positive (excitatory) weights in red and negative (inhibitory) weights in blue. Each unit in the output layer (bottom) predicts the activity of one neuron. The total number of parameters (trainable values) in each layer is listed to the right, with the total number for the model at the bottom left. **E.** Example filter from the first layer of the population CNN model (circled in D). This filter resembles one typically observed in a standard LN model for a single neuron**. F.** Prediction correlation for each unit, where unit number corresponds to row in the population raster plots in B-C.

An example of a 1Dx2-CNN fit to the A1 dataset illustrates the comprehensive nature of the population CNN models (Fig 2). The first layer consists of 70 1D convolutional units that resemble the filters used in standard LN model fits (Fig 2D and 2E). This layer, in conjunction with the 80-unit convolutional layer that follows, defines a space of spectro-temporal channels upon which the subsequent dense layers depend. Filters comprising the input layer resemble traditional LN model fits, and they may provide insight into the general spectro-temporal features involved in auditory coding. However, the final CNN model prediction is a complex nonlinear combination of these inputs, and a more exhaustive analysis of transformations by the entire model may be required to understand filter properties of individual neurons [7,32]. The positive and negative weights connecting these dense layers produce the time-varying output specific to each unit (Fig 2C), which is compared to the actual neural activity (Fig 2B). Prediction correlation varied across the neural population but was close to a maximum value of 1.0 for many units (Fig 2F).

The number of parameters for each layer (Fig 2D) was defined as the total number of trainable values across all computations. For example, the first convolutional layer requires 70 center frequencies and 70 tuning widths for the Gaussian spectral weightings and 1,050 coefficients for the temporal filter bank, making a total of 1,190 parameters. The total number of parameters across all layers is divided by the number of neurons in the dataset to which the model was fit to provide a measure of the model’s complexity (104,835 parameters / 849 neurons = 123 parameters per neuron [43]).

### CNN models consistently outperform LN models

**Fig 3:**
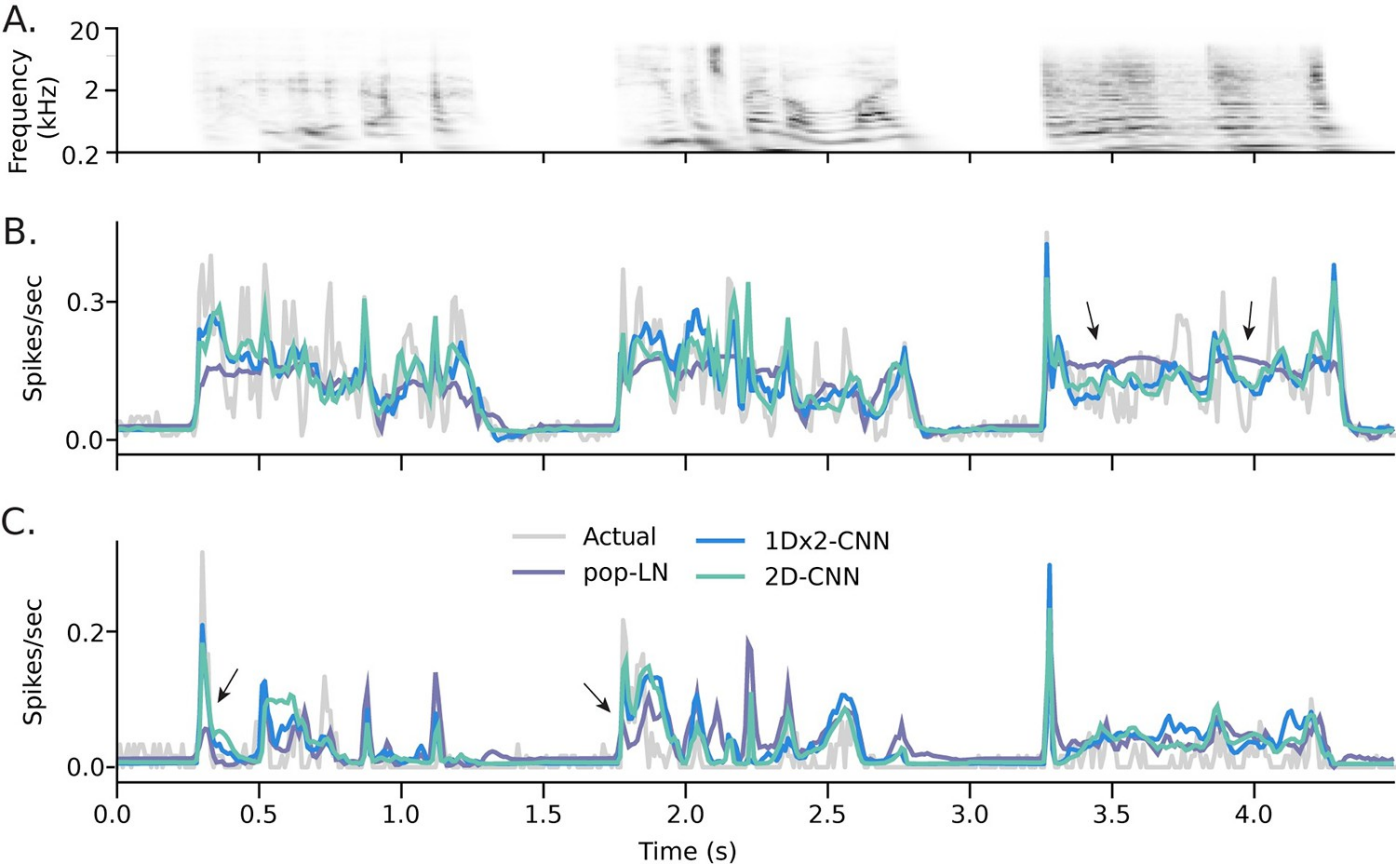
Examples comparisons of actual versus predicted PSTHs for two A1 neurons. **A.** Segment of the stimulus spectrogram from the validation dataset used for testing prediction accuracy. **B.** Actual PSTH (gray) overlaid with the PSTHs predicted by the 1Dx2-CNN (blue), 2D CNN (light green), and pop-LN (purple) models. The two CNN models produced similar predictions that correlated well with the actual response (1Dx2-CNN, r = 0.812, 2D CNN: r = 0.800). The pop-LN model failed to predict many of the temporal features in the actual PSTH (r = 0.590, arrows at 3.5, 4.0 sec). **C**. Actual and predicted PSTH for a second neuron, plotted as in B. Again, both CNN models had higher prediction correlation. Here their accuracy is evident in the observation that they accounted for large transient responses better than the LN model (1Dx2-CNN, r = 0.729, 2D CNN: r = 0.748, pop-LN: r = 0.535; arrows at 0.25, 1.75 sec).

The performance of different model architectures was compared by examining the time-varying PSTH response predicted by different models, fit to data from the same cell. Comparison of example 1Dx2-CNN, 2D-CNN, and pop-LN model predictions suggests that both of the CNN models are better able to capture the dynamics of A1 responses to natural sounds. This is seen especially in their ability to predict the dynamics of transient versus sustained responses following sound onset, which are often not captured by LN models (Fig 3).

To quantify differences in model performance, we fit multiple variants of each model architecture by changing the number of units in one or more layers, which in turn varied the number of fit parameters. Model parameter count provides one means of varying model complexity, as models with more free parameters have greater degrees of freedom in their fits [43]. These manipulations of parameter count also permit exploring the boundaries between architectures. For example, a single-CNN model with one filter in the first layer reduces to a standard LN model. Thus, by varying layer size, we explored a continuous space of models ranging from the LN model to much larger CNNs. For the LN and STP models, we varied the number of fit parameters by changing the rank of the convolutional filter, which can be tuned to optimize performance [3,43].

**Fig 4:**
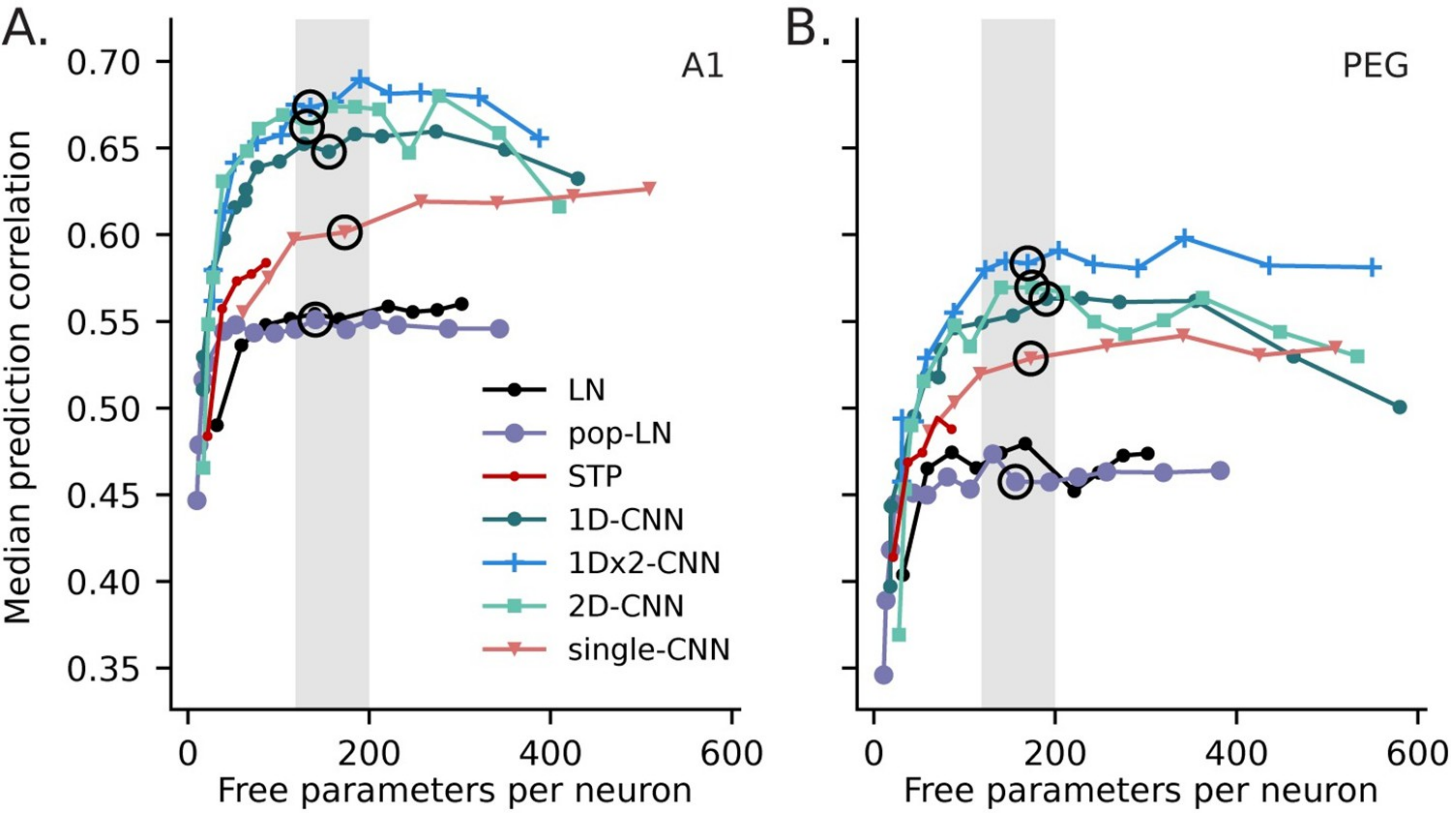
Performance of models from each architecture with variable parameter count. **A**. Pareto plot compares model complexity (parameter count) versus median prediction correlation for each model in A1 (n = 777 auditory-responsive neurons). CNN models maintained consistently higher prediction correlation across a wide range of complexity. Lines connect models in each of the six architectures. For population models, parameter count is normalized by the number of neurons that were simultaneously fit. Circles indicate exemplar models from each category with similar complexity (150–200 parameters per neuron, shaded region), which are examined in more detail in subsequent analyses. **B**. Pareto plot comparing model performance in a secondary field (PEG, n = 339), plotted as in A. Relative performance differences across model types were comparable to A1, but median prediction correlation was lower for all models.

First, we consider the results from A1 data. We compared the performance of models with different architectures and sizes in a Pareto plot (Fig 4A). Prediction correlation increased as model parameter count increased, and it approached asymptotic performance at around 150–200 free parameters per neuron for most architectures. We selected an exemplar model from each architecture with near-asymptotic performance and similar numbers of fit parameters (circled points, Fig 4A). Matching parameter counts provides a means of balancing the signal to noise of model fits. Unless otherwise noted, subsequent analysis focuses on these models and a subset of auditory-responsive neurons, defined as neurons whose activity was predicted above chance by all three of the 1Dx2-CNN, pop-LN and single-CNN exemplar models (p < 0.05; A1: n = 777/849, PEG: n = 339/398).

Median prediction correlation for the LN and pop-LN models was nearly identical, indicating that pooling data across the neural population did not benefit performance of this less complex architecture (signed-rank test between exemplar models; A1: *p* = 0.133, PEG: *p* = 0.167). For subsequent analyses, we focused on the pop-LN model, since there are fewer differences between its architecture and the population CNNs.

**Fig 5:**
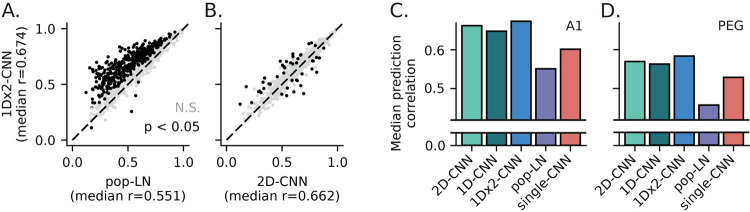
LN and CNN exemplar model performance. **A**. Scatter plot comparing prediction correlation for exemplar pop-LN and 1Dx2-CNN models for each neuron in the A1 dataset. The 1Dx2-CNN model had significantly higher prediction correlation for 376 of the 777 neurons. **B**. Scatter plot comparing the 2D-CNN and 1Dx2-CNN models, plotted as in A. Prediction correlations were comparable in this case, but the 1Dx2-CNN model still represents a small overall improvement (signed-rank test, p = 1.10 x 10^−8^). **C**. Median prediction correlation in A1 for exemplar models representing each architecture. All differences were statistically significant (signed-rank test, 1D-CNN vs. 2D-CNN: p = 9.21 x 10^−3^, other comparisons: p < 10^−7^). **D**. Prediction correlation for PEG data, plotted as in C. Again, the pop-LN model had the lowest prediction correlation (median 0.46). The difference between 1Dx2-CNN and 2D-CNN was not significant (0.58 vs. 0.57, respectively, signed-rank test, p = 0.883), but all other differences were (p < 10^−4^). Although overall prediction correlation was lower for PEG than A1, the relative difference in performance between models was the same for both areas, and the 1Dx2-CNN model was the best-performing in both areas.

When model parameter count was matched (i.e., for exemplar models with approximately the same number of fit parameters per neuron), prediction correlation was higher for all population CNN models (1D-CNN, 1Dx2-CNN, 2D-CNN) than for the LN architectures (Figs 4 and 5). The greater accuracy of CNN models was consistent across the neural population: the 1Dx2-CNN model predicted responses more accurately than the pop-LN model (p < 0.05, jackknifed t-test) for almost half of auditory-responsive A1 neurons (376/777) while the opposite was true for only four neurons (Fig 5**A**). On average, the best 1Dx2-CNN model accounted for 47% of the explainable variance in the A1 data, compared to 31% for the best LN model and 39% for the best single-CNN model (median prediction correlation: 1Dx2-CNN, 0.67; pop-L, 0.55; single-CNN, 0.60).

The explanatory power of the 1Dx2-CNN model was also greater than that of a nonlinear model that explicitly incorporates short-term plasticity and gain control, which accounted for 37% of explainable variance (Fig 4A, [22]). To compare these models with other multi-filter model frameworks, we fit the same data using two published multi-filter model libraries (NIM and iSTAC, S1 Fig, [19,20]). Both libraries consistently converged on stable model fits, but prediction accuracy was lower than the CNN models (median prediction correlation: NIM 3-filter model, 0.52; iSTAC 3-filter model, 0.43; single-CNN, 0.60, n = 775 A1 units). Thus, the CNN-based models performed best among several other previously proposed model architectures.

In addition to their increased accuracy relative to LN models, the population CNN models performed better than the single-CNN model. This result indicates that the improved performance of the population models was not solely due to their neural network architecture but also reflected the gain in statistical power from pooling data across neurons. At the same time, the single-CNN model did outperform both the LN and pop-LN models, confirming a benefit of the CNN architecture over the traditional LN model. The single-CNN architecture also continued to increase in accuracy as parameter count grew, suggesting that a large CNN can indeed be an effective single-neuron model if a sufficiently large dataset is available.

Among population CNN models with similar parameter count, prediction correlation was quite similar, suggesting that the specific architecture was not critical to performance (Fig 5B, 5C). Adding an extra convolutional layer to the 1D-CNN model (Fig 1D), which used filters derived from auditory LN models, did increase prediction correlation (signed-rank test, p = 3.89 x 10^−18^, Fig 5C), making 1Dx2-CNN (Fig 1E) the best-performing model tested. This modest but significant difference suggests that CNN architectures can benefit from modality-specific features like the 1D, temporal-only convolution. Further improvements may be achieved by architectures that incorporate other known properties of the auditory system.

The relationships between models described for A1 also held true for PEG (Figs 4B and 5D). The exemplar 1Dx2-CNN model accounted for 34% of the explainable variance in the PEG data (median prediction correlation 0.58), compared to 21% for the pop-LN model and 28% for the single-CNN model (median prediction correlation 0.46 and 0.43, respectively). Compared to A1, median prediction correlation was consistently lower for PEG across all architectures and model sizes. This lower prediction accuracy is consistent with previous work arguing that non-primary cortex is selective for more complex sensory features, and accurate modeling is likely to require larger fit datasets. The relative improvement of the 1Dx2-CNN model over the pop-LN model was greater in PEG (63% increase in variance explained) relative to A1 (49% increase), consistent with the idea of greater nonlinearity in non-primary cortex.

The relative performance of the CNN models did not depend on the metric used to evaluate prediction accuracy. Previous studies have used log likelihood of the predicted response and mutual information between predicted and actual response to evaluate model performance [16,20]. We compared prediction accuracy between the pop-LN and 1Dx2-CNN models using these alternative metrics and found a similar pattern of improvement (S2 Fig). The 1Dx2-CNN model had higher prediction accuracy across all metrics, and the relative different between metrics was correlated across neurons, suggesting that they measure the same improvements in performance.

### Functional equivalence of CNN models

**Fig 6:**
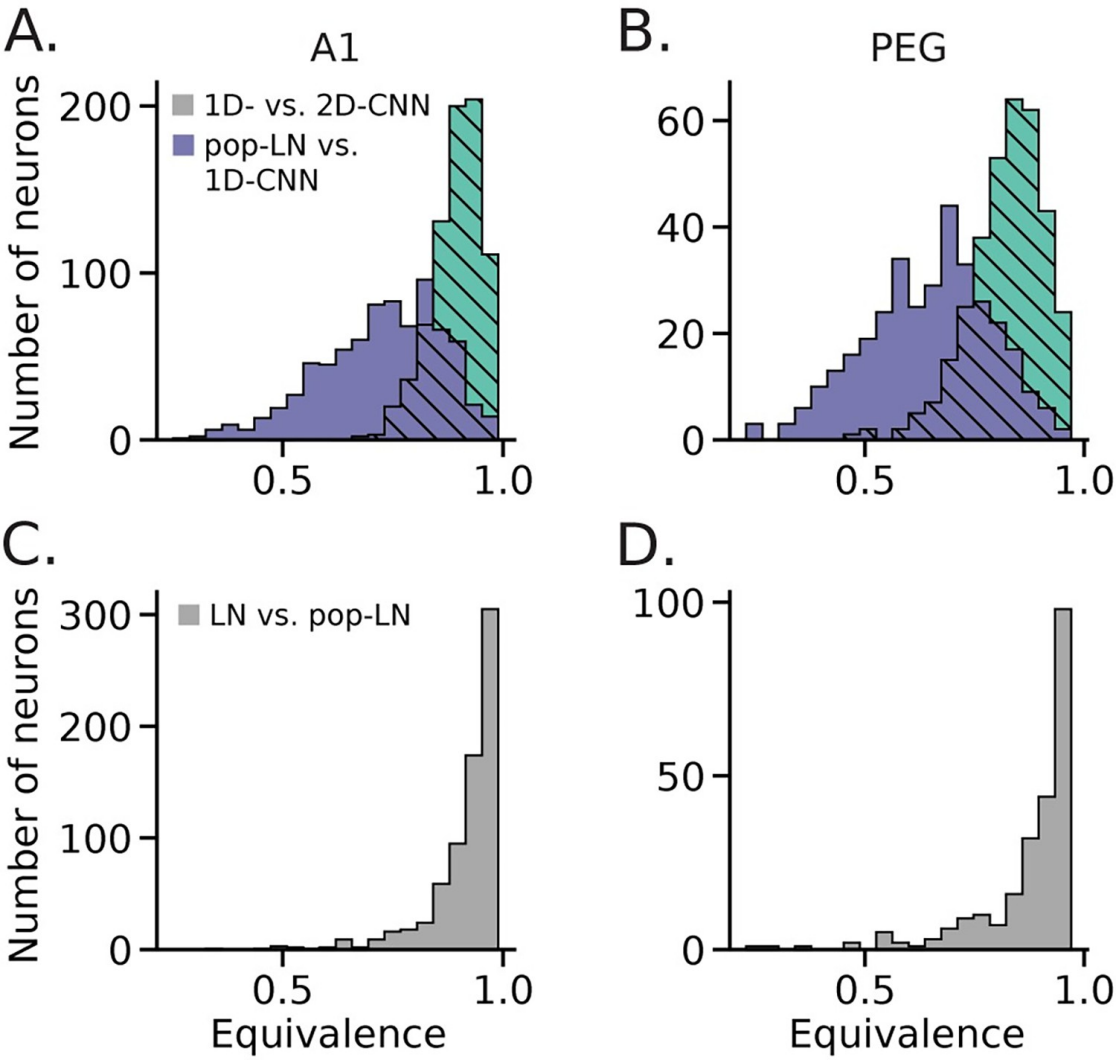
Quantification of equivalence between CNN, pop-LN, and LN exemplar models. **A**. Histogram of equivalence (correlation between predicted PSTHs) on the validation data for auditory-responsive A1 neurons (n = 777/849), between 2D CNN and 1Dx2-CNN models (light green, hatched) and between 1Dx2-CNN and pop-LN models (purple). Equivalence was greater between the two CNN models than between the 1Dx2-CNN and pop-LN models (signed-rank test, p = 1.47 x 10^−128^). This result indicates that CNN models achieved higher prediction accuracy over the LN architectures in similar ways. **B.** As A, but for PEG neurons (n = 339/398). Here again we observed higher median equivalence between the CNN models (p = 1.64 x 10^−55^). **C.** Histogram of equivalence between LN and pop-LN models for A1 neurons. The distribution is shifted even farther toward the 1.0 bound, indicating that the LN and pop-LN models predicted closely matched PSTHs for most neurons. **D.** As C, but for PEG neurons. The equivalence distribution is similarly right shifted.

While the CNN models all predicted neural activity with similar accuracy, it was not immediately clear whether they captured the same functional properties or if their improvements over the LN model instead reflected each one’s ability to account for distinct aspects of neural function. Examples of the detailed dynamics predicted by each CNN model were closely matched between predictions for the same neurons, suggesting that they did capture the same functional properties (Fig 3). To assess equivalence quantitatively, we measured the correlation coefficient between the time-varying activity predicted by the 1Dx2-CNN, 2D-CNN, and pop-LN models (Fig 6).

Equivalence was substantially higher between the two CNN models than between the 1Dx2-CNN and LN models (signed-rank test, p = 1.47 x 10^−128^). Furthermore, the equivalence scores for the CNN models qualitatively resembled what we would expect for truly equivalent models: a right-shifted distribution near the maximum score of 1.0 (Fig 6A and 6B). The LN and pop-LN models served as a useful baseline for this comparison since we expected, and observed, high equivalence for these models based on their architectures (Fig 6C and 6D). Thus, the population CNN models appear to capture the same functional properties for most neurons in the dataset.

### Population models generalize to novel datasets

We hypothesized that, when fit to the activity of many neurons, population models capture an encoding subspace shared across neurons in the brain area that they describe. Whether constrained by information bottlenecks in neural circuits or by developmental plasticity following exposure to behaviorally important sound features, the space of sound representations in cortex is likely to be lower-dimensional than the space of all possible stimuli. Similarly, the shared tuning described by population models is constrained by the dimensionality of the final hidden network layer. We reasoned that if these models captured the actual neural subspace, then they should be able generalize to data from neurons that were not included in the original model fit.

**Fig 7:**
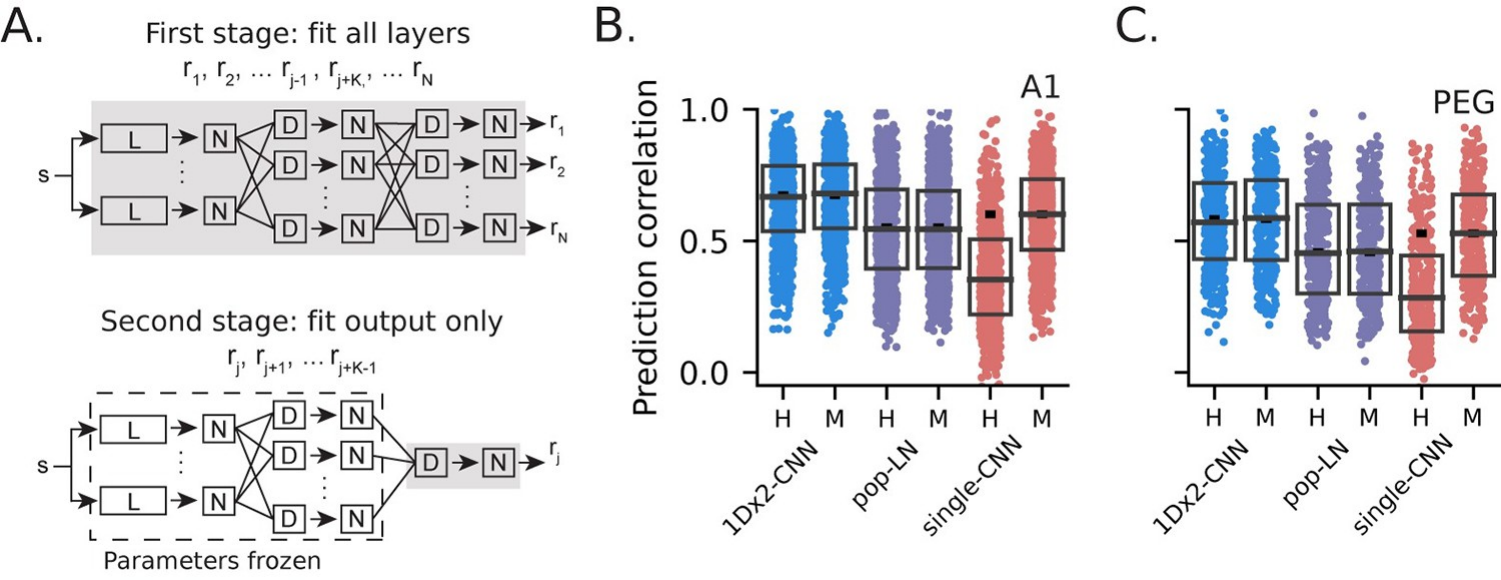
Generalization of population models to novel neural data. **A.** Schematic of two-stage fitting process for held-out and matched models. For the held-out model, responses of all *K* neurons from a recording site were excluded from the first stage fit. For the matched model, *K* neurons from other sites with similar prediction correlation were excluded during the first stage. For both models, all parameters except those in the final layer were frozen (dashed blue box) while individually fitting the *K* excluded responses in the second stage. **B**. Prediction correlation of A1 held-out (H) and matched (M) models. Boxes show the 1st, 2nd and 3rd data quartiles, and the small horizontal dash shows median performance for the full model (Fig 5C). For both population models, the difference in prediction correlation between held-out and matched was not significant (signed-rank test, 1Dx2-CNN: p = 0.751; pop-LN: p = 0.866), indicating that these models generalize well to novel data. In contrast, there was a substantial decrease in prediction correlation for the held-out single-CNN model (p = 4.93 x 10^−81^). **C**. Generalization for PEG neurons, plotted as in B. Once again, performance was the same for held-out and matched population models but significantly decreased for the held-out single-CNN model (signed-rank test, 1Dx2-CNN: p = 0.418; pop-LN: p = 0.941; single-CNN: p = 6.46 x 10^−33^).

To test this hypothesis, we re-fit the 1Dx2-CNN, pop-LN, and single-CNN models using two alternative approaches (Fig 7A). In a “held-out” model, all data from one recording site was excluded during the first stage of the model fit. The output layer was then re-fit to each excluded neuron in the second stage, but model parameters were kept fixed for all preceding layers. In other words, the held-out model was pre-trained on data that excluded both the neuron being predicted and its neighbors from the same recording site. Excluding the entire recording site precluded the possibility of fitting to activity of neurons in the local network with highly overlapping selectivity. As a control, we also fit a “matched” model in which a subset of neurons from other recording sites was excluded during the first stage of fitting, such that the number of neurons excluded was the same as for the held-out model. This design ensured that the amount of fit data was matched to that of the held-out model, and data from the predicted neuron was used to fit all model layers (see Methods).

If performance of the held-out and matched versions of a model is equal for a given neuron, then the held-out model already accounts for the spectro-temporal encoding properties of that neuron in its pre-trained layers. In this case, we can say that the model generalizes well: the response of any novel neuron from the recorded brain area can be accounted for simply by re-fitting the output layer of a pre-trained model. When we compared performance of the held-out and matched versions of the three models (Fig 7B, 7C), we found that the 1Dx2-CNN and pop-LN models both generalized well: there was no difference in prediction correlation between the two fitting strategies (signed-rank test, A1; 1Dx2-CNN: p = 0.751; pop-LN: p = 0.866; PEG; 1Dx2-CNN: p = 0.418; pop-LN: p = 0.941).

Since it was fit to data from a single neuron, it is not surprising that the single-CNN model generalized poorly and did not capture the sensory space encoded by a novel neuron. We note, however, that the held-out single-CNN model did perform above chance. This may reflect a general sensitivity to stimulus onset and offset in auditory cortex that can be captured by a very low-dimensional stimulus space, providing a basic control for the matched versus held-out comparison for the population models.

The finding that the pop-LN model generalized to new data illustrates a broader value of the population modeling approach. The LN model spans a more limited encoding subspace than the CNN models and thus does not predict activity as accurately. Still, this population model captures the auditory space spanned by the LN model, permitting the simpler architecture to generalize to data from new neurons. This observation suggests that the population modeling approach can benefit analysis using any model architecture, including new architectures that outperform those considered in the current study.

**Fig 8:**
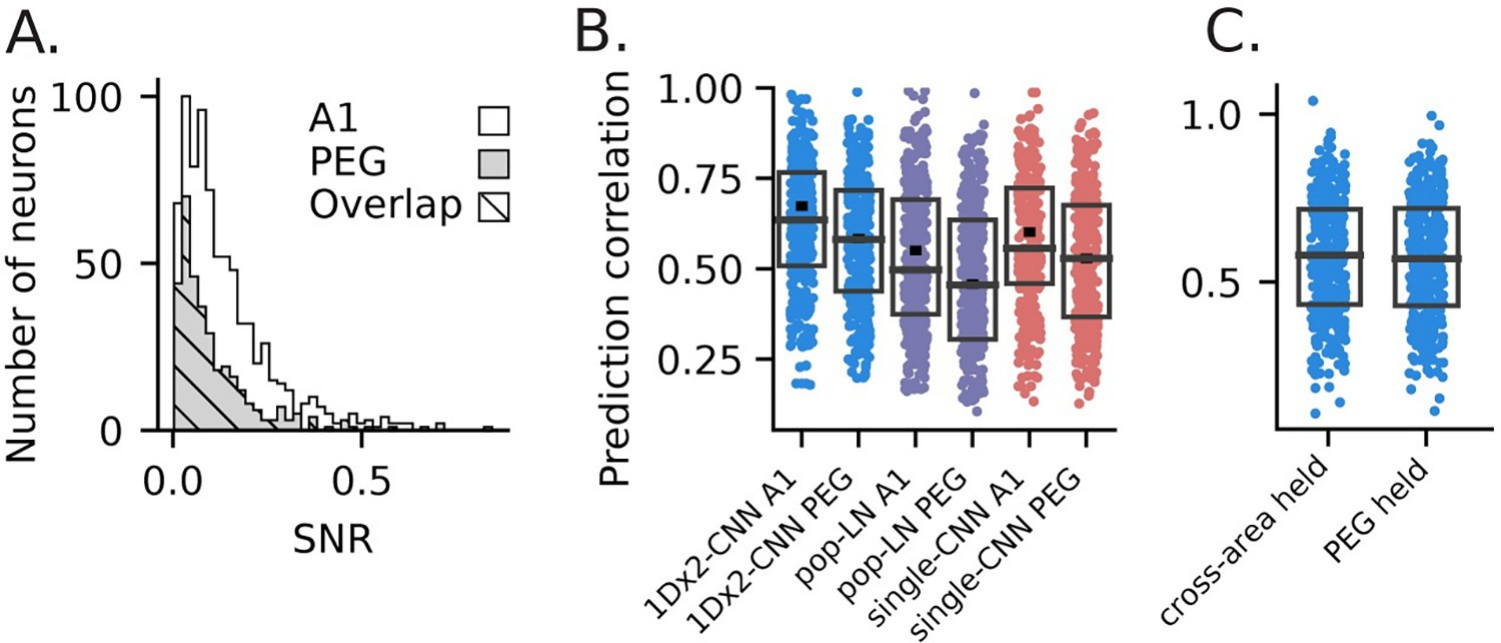
Comparison of model performance between primary (A1) and secondary (PEG) auditory cortical regions after controlling for differences in SNR. **A**. SNR scores for all neurons from A1 (white), PEG (gray), and an overlapping subset (hatched) of neurons with matched distributions. Median SNR was higher for A1 (md = 0.0989, U test: p = 2.49 x 10–6) than for PEG (md = 0.0713), and the A1 subset had a lower median SNR (md = 0.0709) while the median for the PEG subset was mostly unchanged (md = 0.0710). To form the overlapping set, we selected the largest possible subset of neurons from each brain region for which binned distributions of SNR scores for the two subsets were identical. With this approach, the subset was formed primarily by excluding high-SNR neurons from A1 since PEG neural responses were less reliable overall. **B**. Prediction accuracy of three exemplar models for neurons in the A1 and PEG subsets. Boxes show the 1st, 2nd and 3rd quartile for the selected subset, and the small horizontal dash indicates median performance across all neurons from that brain region. The increase in prediction accuracy for A1 was smaller for this subset, but still significant (U test; 1Dx2-CNN: p = 6.685 x 10^4^; pop-LN: p = 6.743 x 10^−4^; single-CNN: p = 4.78 x 10^−4^), indicating that the increased SNR of A1 responses only partially accounts for our models’ higher prediction accuracy for A1 neurons. **C.** Prediction correlation for the 1Dx2-CNN PEG held-out model (Fig 5C, median r = 0.569) and a “cross-area” held-out model (md = 0.581). Pre-fitting to A1 data proved to be just as effective as pre-training on PEG data for predicting PEG neural responses (signed-rank test, p = 0.119).

We also evaluated the ability of models to generalize to other brain regions by fitting a model first to data from A1 and then fitting only the output layer to data from the secondary cortical area PEG (Fig 8). For this comparison, we selected a subset of neurons from A1 that had a matched distribution of auditory responsiveness (SNR) to the set of PEG neurons (Fig 8A). After controlling for this difference in responsiveness, prediction accuracy was still significantly lower in PEG (Fig 8B). To fit the cross-area model, we used the held-out approach (Fig 7A) to pre-train a 1Dx2-CNN model on A1 data, and then fit the output layer using data from PEG neurons in the matched-SNR subset. The important distinction in this case is that the output layer of each model was always fit to data from a PEG neuron, but the earlier layers were fit using an independent set of either A1 or PEG responses. The model fit initially to A1 data performed as well as a model fit initially with PEG data (Fig 8C). This similarity in performance suggests that CNN models generalized across cortical areas, in addition to new neurons in the same area.

### Pre-trained models benefit analysis of smaller datasets

Given the ability of a pre-trained model to generalize to new data, we reasoned that such a model should also be beneficial to the analysis of smaller datasets that measure neural responses to fewer auditory stimuli. The amount of data available from neurophysiological recordings is often limited relative to the large datasets typically required for CNN models. This problem is especially acute in studies of animal behavior, where data is limited by the number of trials an animal is motivated to perform during a single recording session [46]. Thus, a pre-trained model that can accurately describe small datasets acquired in diverse experimental settings would be of broad value to the study of auditory coding.

**Fig 9:**
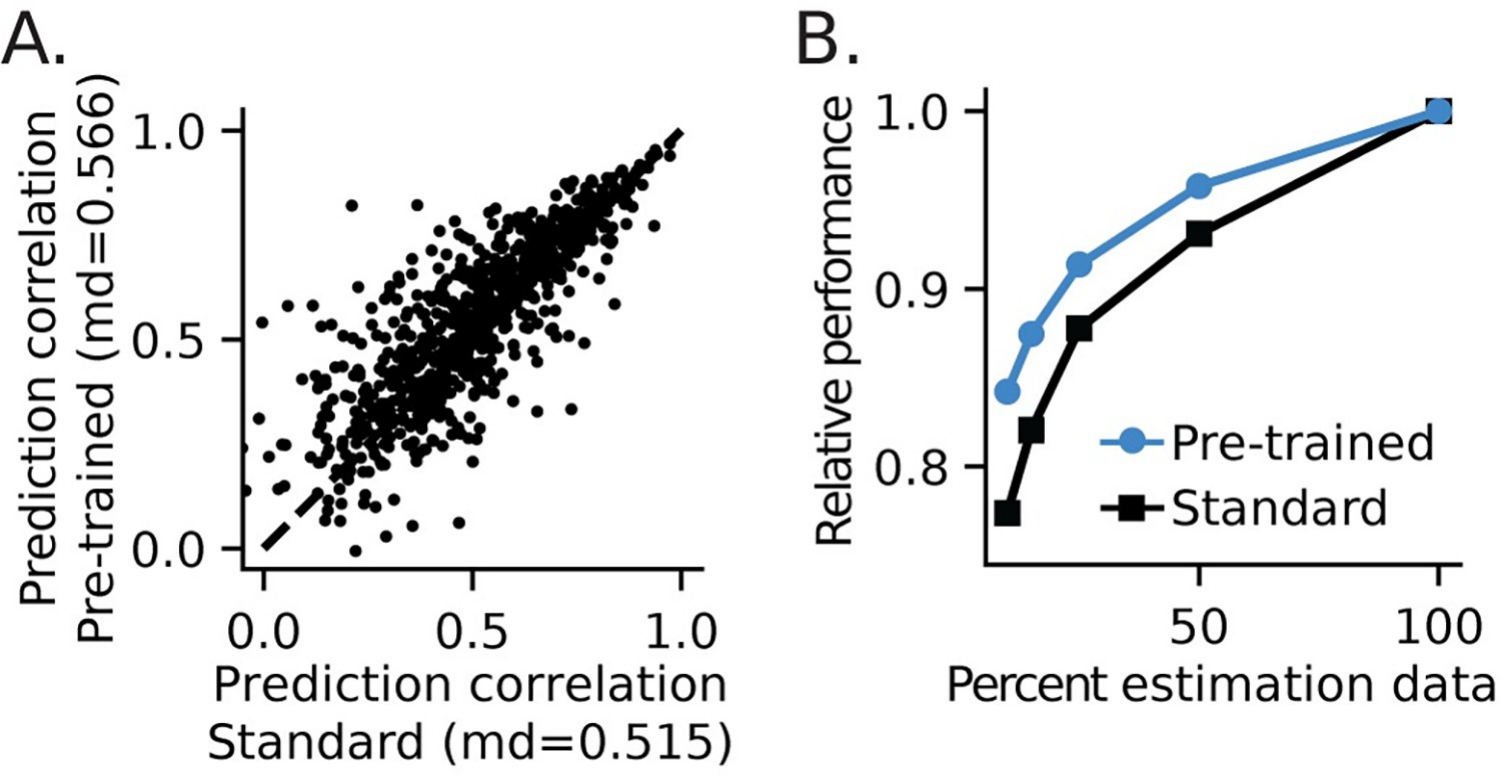
Generalization of a pre-trained 1Dx2-CNN model to smaller datasets. **A.** A pre-trained model was fit to every stimulus with neurons from one recording site excluded. The output layer was then re-fit for the excluded neurons, using a fraction of the available stimuli. The standard model was fit to all neurons, but only a subset of stimuli was used for the entire fit. Scatter plot compares prediction correlation between the pre-trained and standard models using 10% of available data. On average, the pre-trained model more accurately predicted the subsampled data (signed-rank test, p = 2.91 x 10^−21^). **B.** Median prediction correlation for pre-trained and standard models fit to subsampled data, normalized to performance of the model fit to the full dataset. Improved accuracy of the pre-trained model was consistent across all subsample sizes (signed-rank test, p < 10^−9^). Performance converges when 100% of data are used to fit both models.

To test for benefits of generalization on smaller datasets, we subsampled spiking data over 10–100% of the original dataset. A 1Dx2-CNN held-out model was pre-trained on 100% of data from all but one recording site, as above. The output layer was then re-fit to individual neuron responses from the excluded site, using only the subsampled data. In other words, the model drew on a much larger dataset for fitting the initial layers but only used the smaller subsample for fitting the final layer. To prevent bias from potential instability in recordings, the subsampled data were drawn uniformly from trials spanning the entire duration of each experiment. We compared this model to a standard fit, in which both stages of fitting used data from all recording sites, but only a subsample of data was used for the entire fit. The standard model served as a control by representing a scenario in which data quantity is limited by experiment duration or other factors and no pre-trained model is available. Performance of models fit with the smaller datasets was quite variable, but the pre-trained held-out model performed better than the standard model on average (Fig 9A, signed-rank test, p = 2.91 x 10^−21^). The benefit of pre-training extended across all subsamples tested (Fig 9B, signed-rank test, p < 10^−9^).

## Discussion

We developed a convolutional neural network (CNN) architecture that simultaneously models the responses of several hundred neurons to dynamic natural sounds. Consistent with previous work in the visual system [38,39], these population CNNs substantially outperformed traditional LN models of auditory coding, as well as CNNs fit to the activity of individual neurons. Moreover, population models were generalizable: the output layer of a pre-trained model could be re-fit to novel data without a reduction in prediction correlation. The generalizability and improved performance of the population CNN models is consistent with the hypothesis that their early layers describe a comprehensive sensory space, encompassing sound encoding by all neurons in the auditory field being analyzed.

### Population network models provide a natural expansion of linear-nonlinear encoding models

Alternatives to the LN model have been proposed to better characterize auditory encoding. Polynomial expansions, based on Taylor and Volterra series, are the classic extension of linear models to better account for sensory activity [11–13]. Although theoretically well motivated, these second-order models provide only modest improvements over the first-order, linear model. This shortcoming is likely due to two factors. First, second-order models require larger datasets for fitting and are thus more prone to estimation noise than linear models. Second, even with adequate fit data, the expanded functional space of a second-order model may not align well to the actual nonlinear biological properties of auditory neurons. Another alternative class of model attempts to address the latter limitation by accounting for specific biological nonlinearities, such as contrast gain control, short-term plasticity, and local inhibition [3,14,15]. With an empirically targeted design, these models tend to require less data for fitting. However, they still only provide modest improvements to performance and may be limited in their ability to account for the full complement of nonlinearities present in sensory neural responses.

Multi-filter LN models [16–20] and artificial neural networks [7,21] provide a third alternative that can account for a broader range of nonlinear properties. These families of models are all similar in that they project stimuli into a multi-dimensional subspace and then perform a weighted sum across this space to produce a predicted response [13]. These large and complex models have traditionally required specialized fitting procedures to account for sampling limitations and noise in spiking data [21]. In the current study, we showed that the standard LN model architecture can be extended smoothly into small neural networks with convolutional units resembling linear filters in LN models. Moreover, we leveraged statistical power across neurons to fit these models on a modestly sized dataset without highly tuned optimization techniques. Population CNN models fit using this approach performed substantially better than the LN model and other nonlinear models, including models that account for nonlinear synaptic plasticity [3] and existing multi-filter models [19,20]. Analytically, the single-CNN is an instantiation of a multi-filter model. Thus, one might expect similar prediction accuracy to the other multi-filter models. The fact that the single-CNN and the population CNNs performed consistently better may reflect details of the model architecture. Several aspects of the CNN models developed here were chosen based on previous, exhaustive investigation of optimal LN model architectures for sound coding in auditory cortex [43]. Most notably, all the CNN models use low-rank spectro-temporal filters and a double exponential nonlinearity in the final layer. Incorporating these elements into other multi-filter model frameworks could yield increases in their performance.

For all population CNN architectures tested (1D-CNN, 1Dx2-CNN, 2D-CNN), prediction correlation increased as model complexity increased and reached an asymptote at about 150–200 free parameters per neuron. This parameter count reflects an increase in model complexity relative to an efficiently parameterized LN model (< 100 free parameters per neuron), but still falls far short of the approximately 450 free parameters per neuron required for an equivalent full-rank LN model [43]. The comparison between fit dataset size and prediction correlation shows that performance was limited by the amount of fit data available (Fig 9), even for the relatively diverse stimulus set used in this study. Thus, further increasing the amount of fit data should lead to even more accurate model predictions. A more general model fit to a larger dataset will likely require more free parameters.

Surprisingly, there was not a clear “winner” among population CNNs: these models were highly equivalent, and differences in prediction correlation were small. Despite theoretical guarantees that *some* CNN exists that *can* approximate a given neural encoding function, there is no guarantee that any specific CNN of a fixed size can meet that goal. There is also no guarantee that a network’s parameters can be optimized for a particular function algorithmically, even if the chosen architecture is sufficiently large [47]. Accordingly, we had no expectation of equivalence between the CNNs we built despite their similarities. However, our results demonstrate that population CNN models can be fit robustly and produce largely equivalent predictions, regardless of details of the model architecture. At the same time, the best-performing model employed a 1D-CNN architecture, derived from spectro-temporal LN models used in the auditory system. In these models, early layers perform convolution only along the temporal axis and perform a weighted sum across spectral channels. In contrast, the 2D-CNN architecture, which was developed for visual signal processing, performs convolution across both the time and frequency axes [7,27]. This result suggests that CNN architectures might be optimized differently, depending on functional properties known to exist in the system of study. Incorporating additional biological functions into CNN models, such as gain control or short-term plasticity, could also lead to more effective models [48].

### Toward a generalized model of auditory cortex

The goal of a fully generalizable brain model that can simulate neural activity across novel experimental conditions is not new [49]. However, attempts to build detailed models from the ground up have met with limited success, and it remains unclear which details of neural circuits and biophysical mechanisms are required to mimic natural function. In the current study, we took a more functional approach to building a generalizable model. We used nonlinear regression to model the auditory system, without accounting for detailed biological circuitry. In this sense, the population CNN models we developed bear a closer resemblance to foundation models, which have grown increasingly valuable across the field of machine learning [35]. In the domain of language processing, for example, models such as BERT and GPT-3 can be applied to a wide range of language problems with little additional training [36,50]. A foundation model for biological auditory coding could draw on existing foundation models or the architectures developed here to describe a wide range of neurophysiological processes. Previous studies of auditory and visual neurophysiology have promoted a similar idea [27,31]. Intermediate representations in large CNN models, initially fit for a visual or image processing problem, can be used in a generalized linear model to predict neurophysiological responses to sensory stimuli. Here, we found such a model was able to generalize and account not just for sensory selectivity but also for the time-varying response to dynamic natural auditory stimuli.

### Functional differences across the cortical hierarchy

One open question in studies of the auditory system is how sound representations evolve across the cortical processing hierarchy. Traditional LN models have been effective in the brainstem and midbrain [51–53], but their accuracy is limited in auditory cortex [2,3]. Studies comparing multiple cortical fields are limited but indicate LN models perform even more poorly in non-primary auditory cortex than in A1 [41,43]. This is expected since sound-evoked activity in non-primary cortex is modulated more by changes in internal state [41,54] and undergoes complex, long-lasting sensory adaptation that limits the efficacy of encoding model analysis [55]. In this study, CNN models were able to achieve a substantial improvement in performance over the LN model in a non-primary field (PEG). However, prediction correlation was consistently lower for PEG than for A1, even after accounting for differences in response reliability (SNR) between areas. This difference is consistent with the idea that PEG neurons exhibit more selective, nonlinear response properties than A1. In the absence of data limitations, a CNN or related model should be able to account for this sensory selectivity. Thus, the difference in performance indicates that a larger fit dataset is required to characterize PEG neurons as accurately as A1 neurons.

### Neural population dynamics and the space of sensory representation

Questions around the dimensionality of cortical sensory coding spaces are an active area of research [56]. Studies using multi-filter models have argued that the functional properties of single neurons are best described as spanning a sensory subspace [13,16–20]. The population models used here extend this idea to a shared subspace across the entire set of neurons studied. The fact that population CNNs generalize readily to novel neural data suggests that, embedded in their parameters, is a complete space of the sound features encoded across the entire cortical field. The nonlinear combinations of spectro-temporal sound features that comprise each channel of a population model’s final hidden layer may be dynamic and complex. However, the dimensionality of this final layer determines how many channels are recombined to predict the activity of any neuron. The best-performing 1Dx2-CNN model relied on 100 channels in this layer, and the performance of the held-out models indicates that the constrained space this layer represents could account equally well for the activity of any neuron in A1. This result suggests that much of the sensory activity of the many thousands of neurons in auditory cortex can be accounted for by a relatively low-dimensional space.

As high channel-count recordings in neurophysiological research continue to become more prevalent and grow in scale, the feasibility and value of population models will also increase. We expect these benefits to be particularly strong in circumstances where dataset size is constrained by experimental design, such as in studies of behavior. When behavioral factors such as motivation to perform a task limit the number of trials during recordings, statistical power can be increased by recording from a large number of neurons. In addition, sensory coding properties can be considered in a constrained sensory subspace by using a model pre-trained on a larger dataset.

## Methods

### Ethics statement

All procedures were approved by the Oregon Health and Science University Institutional Animal Care and Use Committee and conform to standards of the Association for Assessment and Accreditation of Laboratory Animal Care (AAALAC).

### Data collection

Prior to experiments, ferrets (*Mustela putorius furo*, n = 5) were implanted with a custom steel head post to allow for stable recording. While under anesthesia (ketamine followed by isoflurane) and under sterile conditions, the skin and muscles on the top of the head were retracted from the central 3 cm diameter of skull. Several stainless-steel bone screws (Synthes, 6 mm) were attached to the skull, the head post was glued on the mid-line (Charisma), and the site was covered with bone cement (Charisma and/or Zimmer Palacos). After surgery, the skin around the implant was allowed to heal. Analgesics and antibiotics were administered under veterinary supervision until recovery.

After animals recovered from surgery and were habituated to a head-fixed posture, a small craniotomy (approximately 0.5 mm diameter) was opened over A1 or the secondary auditory field, PEG, immediately ventro-anterior to A1 [40,41]. Neurophysiological activity was recorded using silicon multielectrode arrays (UCLA probes [57]). The array was inserted approximately normal to the cortical surface using a microdrive (Alpha-Omega Engineering EPS). Electrophysiological activity was amplified and digitized (Intan RHD-128) and recorded using open-source data acquisition software (OpenEphys). Recording site locations were confirmed as being in A1 or PEG based on tonotopy, frequency tuning and response latency [40,41].

Single- and multi-unit spiking events were extracted from the continuous, multichannel electrophysiological traces using Kilosort 2 [58]. Units were only kept for analysis if they maintained isolation and a stable firing rate over the course of the experiment. Unit isolation was quantified as the percent overlap of the spike waveform distribution with neighboring units and baseline activity. Isolation > 95% was considered a single unit, and isolation > 85% with < 15% change in spike rate between the first and last quarter of the recording was considered a multi-unit (single units: A1, 567/849; PEG, 314/398). There was no significant difference in median prediction correlation for any of the exemplar models between these groups in either brain area (U test, p > 0.05). Thus, we pooled single- and multi-unit data into a single population for this study, and we refer to these units as “neurons.”

Stimulus presentation was controlled by custom software written in Matlab (Mathworks, R2017A). Digital acoustic signals were transformed to analog (National Instruments PCI6259) and amplified (Crown D-75a). Stimuli were presented through a flat-gain, free-field speaker (Manger) 80 cm distant, 0-deg elevation and 30-deg azimuth contralateral to the neurophysiological recording site. Prior to experiments, sound level was calibrated to a standard reference (Brüel & Kjær). Stimuli were presented at 60–65 dB SPL (peak-to-peak amplitude).

### Natural sound stimuli

Data were collected during presentation of a library of natural sounds (595 1-sec samples, 0.5 sec ISI). Approximately 15% of these sounds were ferret vocalizations and environmental noises in the animal facility, recorded using a commercial digital recorder (44-KHz sampling, Tascam DR-400). Recordings included infant calls (1 week to 1 month of age), adult aggression calls, and adult play calls. No animals that produced the vocalizations in the stimulus library were used in the current study. The remaining 85% of sounds were drawn from a library of human speech, music and environmental noises developed to characterize natural sound statistics [59]. Activity was recorded during a single presentation of 577 samples and 20 repetitions of the remaining 18 samples. The low-repetition data were used for model estimation and the high-repetition data were used for model validation.

### Modeling framework

For all analyses used in this study, spike data for each neuron was converted into a peristimulus time histogram (PSTH), *r*(*t*), the time-varying spike rate, sampled at 100 Hz (10 ms bins). The input to each model consisted of a sound waveform converted into a spectrogram, *s*(*f*,*t*), using log compression and a second-order gammatone filter bank to account for the action of the cochlea [60]. This step was fixed for each model rather than fitting the spectrogram’s parameters, since we have observed little benefit from this additional complexity [43]. The filter bank included *F* = 18 filters with *f*_*j*_ spaced logarithmically from *f*_low_ = 200 to *f*_high_ = 20,000 Hz (approximately 1/3 octave per bin). The filter bank output was downsampled to 100 Hz to match the sampling of the neural PSTH. Filter shapes in the subsequent model definitions correspond to these 1/3-octave frequency bins and 10 ms time bins. Since sampling resolution and spectral resolution were fixed for this study, it is uncertain how higher resolution data might affect these results. However, from past work with the LN model we would expect that increasing either resolution would reduce prediction correlations consistently for all models such that their performance relative to one another would be unchanged [43].

#### Linear-nonlinear models

The linear-nonlinear spectro-temporal receptive field (LN) model is widely used in studies of neural auditory coding [13,61,62], and was used as a baseline for this study (Fig 1A). The first stage of the LN model convolves a finite impulse response (FIR) filter, *h*, with the stimulus spectrogram to generate a linear firing rate prediction, *r*_lin_:

$$r_{lin}(t)=\sum_{f}^{F}\sum_{u}^{U}h_{f,u}\;s(f,\;t-u)\;\;$$

For the models used here, the filter consists of *F* = 18 spectral channels and *U* = 25 temporal bins. In principle, this transformation can be achieved with a single 18x25 filter. In practice, the filter was implemented as a rank D factorization: projection onto an 18xD spectral weighting matrix specified by a Gaussian function followed by convolution with a Dx25 temporal filter, where D varied from 1 to 11. This implementation substantially reduces the number of free parameters without sacrificing model performance [43].

A static sigmoid nonlinearity that mimics spike threshold and firing rate saturation is applied to the result of this convolution to produce the final model prediction. For this study, we used a double exponential nonlinearity:

$$r(t)=b+a\;exp[-exp(k(y_{lin}(t)-s)]$$

where the baseline spike rate, saturated firing rate, firing threshold, and gain are represented by *b*, *a*, *s* and *k*, respectively [43]. This model predicts the activity of each neuron independently from the rest of the recorded data, but we implemented it in TensorFlow using custom layers such that we could run a single “model” for the full neural population [63]. This approach enabled a dramatic speedup in fit time compared to a one-fit-at-a-time strategy and allowed us to use the same optimization routine as for the population models.

#### Single-neuron CNN models

Single-neuron CNN models (single-CNN, Fig 1C) used in this study have two layers. The first layer is a 1D convolutional layer composed of many units that each apply the following operations in series: multiply Gaussian-distributed spectral weights with the spectrogram to produce a single channel, convolve this weighted channel with a rank 1, 250ms temporal filter, then apply an offset rectified linearity (ReLU) to the output of the convolution. The number of units in this layer determines the model’s size. We fit a total of six model variants in the current study, with unit count ranging from 2 to 18. The second layer consists of a single dense weighting unit with a double exponential nonlinearity as the activation function. The output of this unit is the model’s prediction of a single neuron’s time-varying firing rate.

The activation functions described here were also used for all other neural network models in this study: offset ReLU for all intermediate dense and convolutional layers unless otherwise specified, and double exponential (as described for the LN model) for the final dense layer (output).

#### Population CNN models

Population models are derived from the premise that neural populations cooperatively encode sensory information within a common subspace [6,64]. We implemented four population CNN architectures: a single 1D convolutional layer with no activation function followed by one dense layer (pop-LN, Fig 1B), a single 1D convolutional layer followed by two dense layers (1D-CNN, Fig 1D), two 1D convolutional layers followed by two dense layers (1Dx2-CNN, Fig 1E), and three 2D convolutional layers followed by two dense layers (2D-CNN, Fig 1F). For all population models, the number of units in the output layer is equal to the number of neurons in the population used for fitting (n = 849 for A1, n = 398 for PEG).

The pop-LN model resembles the LN model, in that the encoding properties of a single neuron can be collapsed into an LN model. The only difference is that the entire population shares a subspace defined by the convolutional layer, while weights for individual neurons are only computed in the final layer. We consider this design to be the minimal change needed to convert the single-neuron LN model into a population model. We tested 15 model variants within this architecture, where the number of convolutional units for each variant ranged from 4 to 300. Note that while the pop-LN model is technically a CNN, we refer to it as the LN model when contrasting it with the CNN models that contain intermediate nonlinearities.

The 1D-CNN model is similar to the single-CNN model, except there is a hidden dense layer after the convolutional layer. We compared sixteen model variants for this architecture, with 5 to 230 convolutional units and 10 to 300 hidden units.

The 1Dx2-CNN model is akin to the 1D-CNN model but has two consecutive convolutional layers instead of one. The first layer uses 150ms filters while the second uses 100ms filters, yielding the same 250ms total “context memory” as the models described above. We fit a total of 14 model variants for this architecture. The number of units in the first and second layers varied from 5 to 150 and 10 to 200, respectively, and the number of units in the hidden dense layer ranged from 20 to 250.

The 2D-CNN model also resembles the 1D-CNN model, but the 1D convolutional layer is replaced with three consecutive 2D convolutional layers each using ten 3x8 filters, encompassing a cumulative 240ms of context memory. We compared 15 model variants for the 2D-CNN architecture, where the number of units in the hidden dense layer ranged from 4 to 300.

To choose the structure of each model, we built progressively larger models out from the canonical single-neuron LN model by hand-selecting models with increasing size and number of network layers. For each model type, we evaluated a small number of wide-ranging hyperparameter combinations like learning rate and early stopping criteria, selected a combination that provided stable fits and good performance for all architectures, and then varied convolutional filter count and/or dense unit count to produce a continuum of model sizes. This was not an exhaustive exploration of the possible hyperparameters for the model architectures chosen, nor did we dive deeply into the full range of possible architectures. Additionally, we chose not to explicitly imitate biological nonlinearities like short-term plasticity or gain control within the models since the relatively brief stimuli used for this study were not designed to probe these slower response dynamics.

### Model optimization

Model parameters were fit using TensorFlow’s implementation of the Adam algorithm for stochastic gradient descent, using a mean squared error (MSE) loss function [44,63]. Loss was computed for all neurons in the population simultaneously. Mean squared error was chosen because we have found in practice it is more well-behaved than popular alternatives like maximum likelihood or mutual information for our analyses, likely because we do not control stimulus statistics or noise distribution. Past work has also demonstrated that several such loss functions produce the same relative model ranking for a given set of parameters, so our choice of loss function is not likely to influence the results of this study [43]. To mitigate overfitting, an early stopping criterion was set using twenty percent of the estimation data. We also tested dropout and L2 regularization [65,66], but we found little to no benefit and chose to exclude them from this study for simplicity.

Models were fit in two stages. For the first stage, models were fit to all units from one brain region simultaneously (n = 849 A1; n = 398 PEG). Parameters were randomly initialized nine times, with a tenth initialization set to the mean of the parameter distributions. Each initialization was used as the starting point for the first of three optimization steps. First, we fit a submodel defined by excluding the output layer while keeping the model otherwise identical. Subsequently, these models were fit with the output layer included but parameters of all other layers fixed. The best-performing initialization was then used as the starting point for a subsequent fit of all model parameters simultaneously. In past studies we found that this heuristic approach, which for the LN model amounts to fitting the linear and nonlinear portions separately, improved single-neuron model performance [22,43]. The same proved to be true for this study’s population models. Most models fit using the same architecture but different initial conditions converged on equivalent fits with very similar performance, while a small number converged in local minima and had much worse performance (10–30% of fits).

In the second stage, the output layer of each model was re-fit to one unit at a time while parameters for earlier layers were kept fixed. The second stage served two purposes. First, it provided a modest increase in performance for population models by optimizing a separate loss function for each unit, rather than a single loss function produced by an unweighted average across neurons. Second, it ensured there were no differences in fitting process for the models in the generalization analyses.

For the test of generalization, the same two-stage procedure was used but with subsets of the data excluded. In held-out models, all K neurons from one site, S, were excluded from the first stage fit. Model parameters from this fit were used to initialize second stage fits for the K excluded neurons. In the case of the matched model, K neurons from other sites with prediction scores similar to those in site S (for a single-neuron LN model) were excluded during the first stage. Thus, the matched model provided a control for the generalization test, in that the number of neurons used for fitting this model was the same as for the held-out model. This method was repeated for every recording site to generate held-out and matched model predictions for every neuron.

### Noise-corrected prediction correlation to evaluate model performance

After fitting was complete, model prediction accuracy was measured on a separate validation dataset as the correlation coefficient (Pearson’s *R*) between the time-varying model prediction and actual PSTH response to the validation stimulus. The PSTH was averaged across 20 repetitions of the validation stimulus, which reduced noise from trial-to-trial response variability. However, some noise from finite sampling remained, and the practical limit on the correlation coefficient measured directly with the PSTH was less than the theoretical maximum of 1.0.

The effect of noise in the validation response can be compensated for by normalizing the measured correlation coefficient by the trial-to-trial response correlation (TTRC) [43,45]. If we define TTRC as the mean correlation coefficient between all unique trial pairs, i ≠ j,

$$\mathrm{TTRC}={\langle \mathrm{corr}(r_{i}(t),r_{j}(t))\rangle }_{i,j}$$

then the corrected prediction correlation is the mean correlation between the predicted, *p*(*t*), and the single-trial actual response, *r*_*i*_(*t*), normalized by the TTRC,

$$R_{\mathrm{norm}}=\frac{1}{\sqrt{\mathrm{TTRC}}}{\langle \mathrm{corr}(r_{i}(t),p(t))\rangle }_{i}$$

For very small TTRC or for small number of repetitions, this approximation can be unstable, but for the 20-repetition validation datasets in the current study, it was stable, adjusting prediction scores by a median of 35%. Importantly, applying this correction allowed for bounded measures of prediction accuracy, but it did not affect relative model performance, as the same correction was applied to every model prediction for the same neuron.

### Alternative multi-filter model frameworks

We compared performance of the models developed in this study to two existing multi-filter model frameworks. The Nonlinear Input Model (NIM) is an extension of the generalized linear model (GLM), which fits two or more linear filters that are separately rectified and then linearly summed and passed through a static sigmoidal nonlinearity to produce a final predicted response [20]. NIM models were optimized by maximizing log-likelihood of the predicted response given the actual response. Hyperparameters were selected using grid search on a subset of the data. Default values were used, except the L1 norm was set to lambda = 5. NIM models with 2 or 3 input filters were tested.

The information-theoretic Spike-Triggered Average and Covariance model (iSTAC) architecture optimizes multiple linear filters using mutual information between the filter outputs and time-varying spike rate [19]. Outputs of the linear filters were the combined through a second-order polynomial nonlinearity optimized using a log-likelihood cost function. iSTAC models were fit with three linear filters.

### Exemplar models

After our exploration of a wide range of model sizes (Fig 3A, 3B), subsequent analyses focused on exemplar models from each architecture. These exemplars were chosen such that each had similar complexity, and that complexity was as low as possible while keeping prediction correlation near the observed asymptote. Architectural hyperparameters were as follows, in layer-order, where N represents the number of neural responses in the data:

- LN: 1 1D convolutional unit (rank-5 factorization), 1 output unit.
- pop-LN: 120 1D convolutional units (250 ms), N output units.
- single-CNN: 6 1D convolutional units, 1 output unit.
- 1D-CNN: 100 1D convolutional units (250 ms), 120 hidden dense units, N output units.
- 1Dx2-CNN: 70 1D convolutional units (150 ms), 80 1D convolutional units (100 ms), 100 hidden dense units, N output units.
- 2D-CNN: 3 layers of 10 2D convolutional units each, in series (each 80 ms x 3 spectral bins, approximately 1 octave), 90 hidden dense units, N output units.

### Model equivalence

To quantify prediction similarity between models, we computed model “equivalence” on the validation data as the Pearson correlation coefficient between PSTHs predicted for each neuron. This approach enables a relative comparison: if one distribution of equivalence scores is shifted farther right, then that pair of models is more similar than the pair of models with the leftmost distribution. This analysis was a variant of a previous method that computed the partial correlation between PSTHs, relative to the prediction of a baseline model [22]. The simpler comparison used in this study introduces the limitation that it cannot be meaningfully applied to models that explain a high amount of variance in the data. In that case, the model predictions *must* be highly correlated with each other, so a separate baseline model would be required to measure their similarity.

### Statistical methods

For all statistical comparisons, we used non-parametric tests since the distributions of the relevant variables were non-normal and no other distribution was apparent. For paired tests, e.g., comparing prediction correlations between different models on the same set of neurons, we used a two-sided Wilcoxon signed-rank test (referred to as “signed-rank test”). For non-paired tests, we used a two-sided Mann-Whitney U test (“U test”). Statistical significance was assigned for p < 0.05. Full p-values are reported for completeness unless many tests are reported simultaneously. Note that due to the large number of units in the recordings, the p-values reported here are often extraordinarily small. These values reflect the relatively large number of neurons in the dataset, which provided substantial statistical power.

To determine whether a model’s prediction was above chance for a given neuron, or if one model’s prediction was significantly more accurate than another’s for that neuron, we used a jack-knifed t-test. For the population analyses reported in Figs 3–9, we only included data from neurons for which the 1Dx2-CNN, pop-LN, and single-CNN exemplar models all performed above chance. We considered neurons that met this criterion to be auditory-responsive (n = 777/849 A1 neurons, n = 339/398 PEG neurons) and assumed the excluded neurons were non-auditory.

### A toolbox for systematic comparison of encoding models

All models in this study were fit using the Neural Encoding Model System (NEMS, https://github.com/LBHB/NEMS). We developed this open-source software package to be a flexible and extensible tool for fitting models to sensory neurophysiology data. Implementing all models in a common framework helped eliminate potentially problematic differences in our analysis pipeline, like optimization routine and cost function evaluation, that otherwise might arise. We will continue expanding the model architectures supported by NEMS, and we invite contributions and suggestions to make this software helpful to the broader neuroscience community.

The complete experimental dataset and illustrative code for loading data and fitting models are available open-access for download (Zenodo, [67]).

## Supporting information

S1 Fig Performance comparison with other multi-filter model frameworks. **A.** Pareto plot shows median performance of model architectures from the current study (repeated from Fig 4) and of multi-filter models estimated using previously published software. The Nonlinear Input Model with 2 or 3 filters (NIMx2, NIMx3, [20]) showed a small improvement over a baseline generalized linear model (GLM, *p*<1e-6, signed rank test). Free parameter counts were higher than for CNN-single because full rank filters were used. Performance was lower than for the CNN models. The information-theoretic Spike-Triggered Average and Covariance model (iSTAC, [19]) with 3 filters required a similar number of parameters as NIMx3, and performance was lower than the other models (n = 775 units with significant auditory response and successful fits for all model frameworks). **B.** Comparison of model performance for PEG, plotted as in A, shows a similar pattern as A1 (n = 337 units with significant auditory response and successful fits for all model frameworks). **C.** Scatter plots compare prediction correlation for GLM versus NIMx3 model (left) and NIMx3 model versus pop-LN (middle) and 1Dx2-CNN models (right). Median prediction correlation for each model is indicated in the x- and y-axis labels and is always significantly greater for the model on the y-axis (*p*<1e-6, signed rank test). While the pop-LN and 1Dx2-CNN models performed better than NIMx3, relative performance across cells was correlated, indicating that both frameworks account for similar auditory activity (correlation between prediction correlation, *r*, indicated in each subplot). **D.** Comparison of prediction correlation for iSTAC versus pop-LN or 1Dx2-CNN models, plotted as in C. Median performance of both pop-LN and 1Dx2-CNN models was higher than iSTAC (*p*<1e-6, signed rank test), but relative performance between iSTAC and the other models was correlated across neurons.
(TIF)

S2 Fig Comparison of model performance metrics. Performance assessed by prediction correlation, mutual information (MI) and log likelihood (LL) shows consistent increases in prediction accuracy for CNN models. **A.** Box plot compares 25-th, 50-th, and 75-th percentile performance of pop-LN and 1Dx2-CNN models across the A1 population (n = 777 auditory responsive neurons, data from Fig 5). Median prediction correlation of the 1Dx2-CNN model is significantly greater than the pop-LN model (signed-rank test, *p* value at top of panel). **B.** Comparison of model performance as measured by MI between predicted and actual time-varying activity [16], plotted as in A. Median MI is significantly greater for the 1Dx2-CNN model. **C.** Comparison of model performance as measured by LL of actual activity given predicted activity, plotted as in A. Median LL is significantly greater for the 1Dx2-CNN model. **D.** Scatter plot compares difference in prediction correlation between 1Dx2-CNN and pop-LN models against the difference in MI for each A1 neuron. There was some variability across individual neurons, but the difference was correlated across the population (Pearson’s *r* and *p* value from Student’s *T*-test at top of panel). The observation that the change in performance is similar across neurons for both metrics is consistent with the idea that the capture similar aspects of model performance. **E.** Scatter plot compares difference in prediction accuracy as measured by prediction correlation and log likelihood, plotted as in D. Again, the change in is correlation across neurons between metrics.
(TIF)
