# Supplementary figures and images for: A convolutional neural network provides a generalizable model of natural sound coding by neural populations in auditory cortex
Source: PLoS Comput Biol. 2023 May 5;19(5):e1011110. doi: 10.1371/journal.pcbi.1011110 (PMC10191263; doi:10.1371/journal.pcbi.1011110)

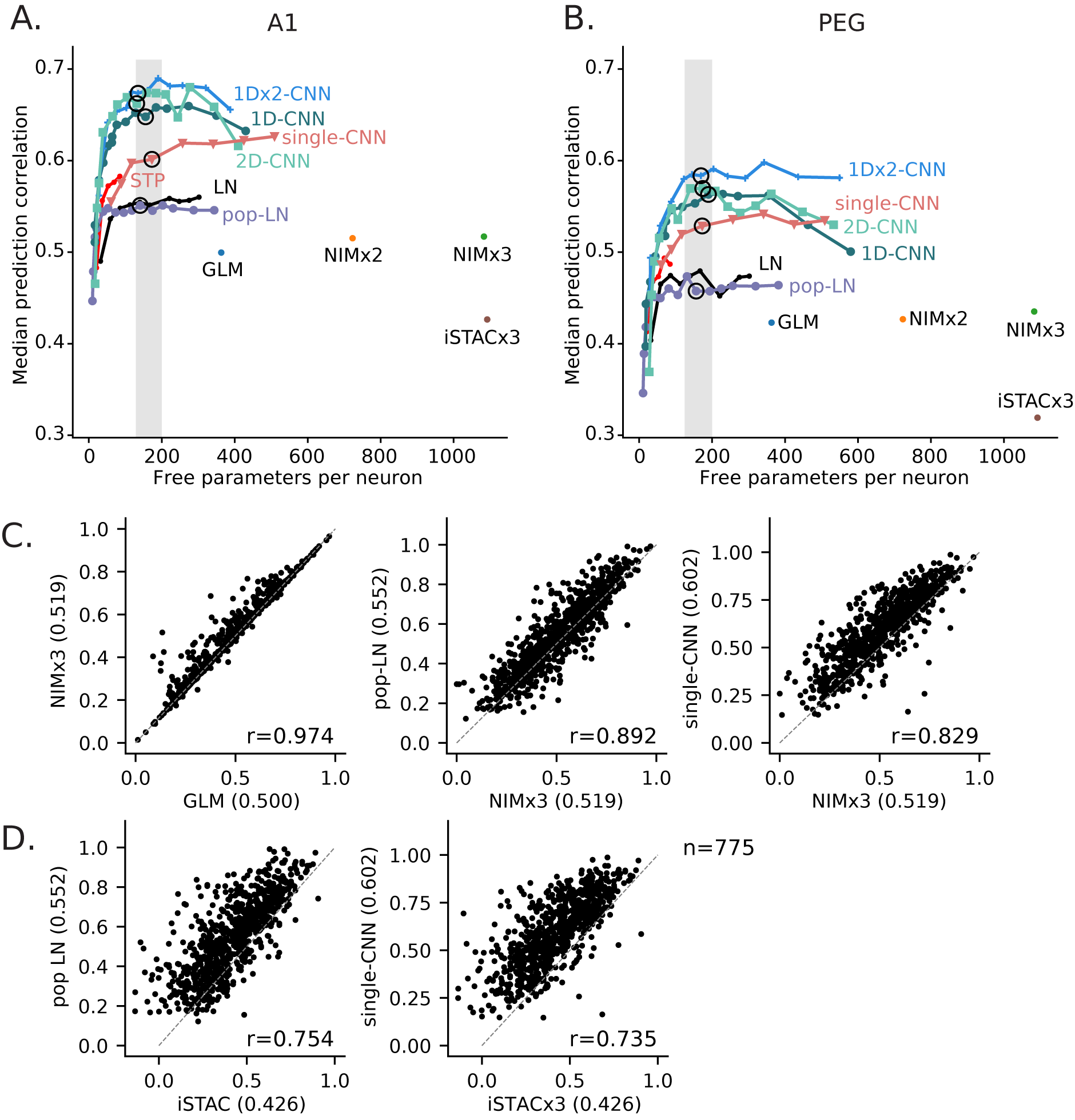

Supplement: S1 Fig — A. Pareto plot shows median performance of model architectures from the current study (repeated from Fig 4) and of multi-filter models estimated using previously published software. The Nonlinear Input Model with 2 or 3 filters (NIMx2, NIMx3, [20]) showed a small improvement over a baseline generalized linear model (GLM, p<1e-6, signed rank test). Free parameter counts were higher than for CNN-single because full rank filters were used. Performance was lower than for the CNN models. The information-theoretic Spike-Triggered Average and Covariance model (iSTAC, [19]) with 3 filters required a similar number of parameters as NIMx3, and performance was lower than the other models (n = 775 units with significant auditory response and successful fits for all model frameworks). B. Comparison of model performance for PEG, plotted as in A, shows a similar pattern as A1 (n = 337 units with significant auditory response and successful fits for all model frameworks). C. Scatter plots compare prediction correlation for GLM versus NIMx3 model (left) and NIMx3 model versus pop-LN (middle) and 1Dx2-CNN models (right). Median prediction correlation for each model is indicated in the x- and y-axis labels and is always significantly greater for the model on the y-axis (p<1e-6, signed rank test). While the pop-LN and 1Dx2-CNN models performed better than NIMx3, relative performance across cells was correlated, indicating that both frameworks account for similar auditory activity (correlation between prediction correlation, r, indicated in each subplot). D. Comparison of prediction correlation for iSTAC versus pop-LN or 1Dx2-CNN models, plotted as in C. Median performance of both pop-LN and 1Dx2-CNN models was higher than iSTAC (p<1e-6, signed rank test), but relative performance between iSTAC and the other models was correlated across neurons. (TIF) [file pcbi.1011110.s001.tif]

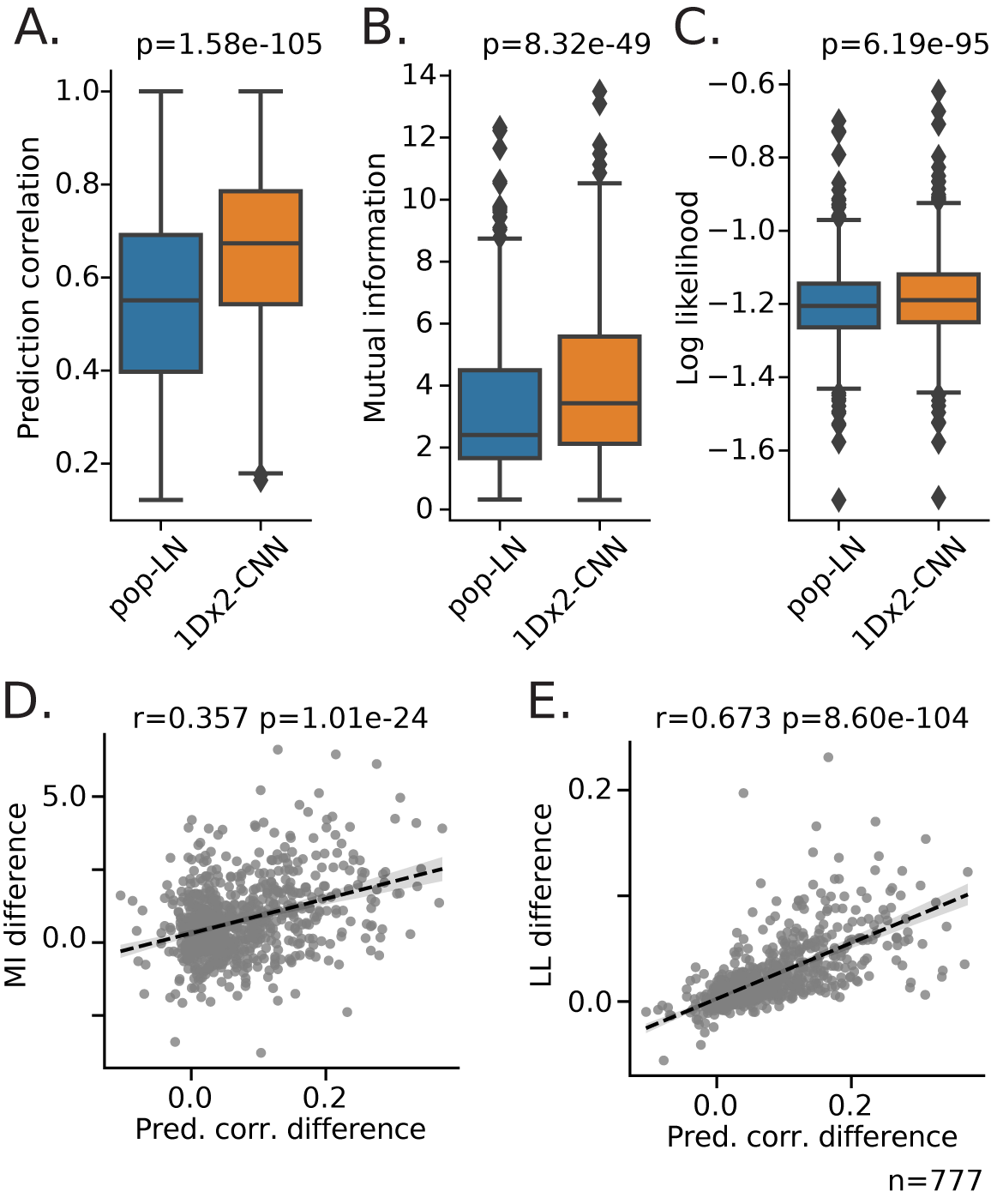

Supplement: S2 Fig — Performance assessed by prediction correlation, mutual information (MI) and log likelihood (LL) shows consistent increases in prediction accuracy for CNN models. A. Box plot compares 25-th, 50-th, and 75-th percentile performance of pop-LN and 1Dx2-CNN models across the A1 population (n = 777 auditory responsive neurons, data from Fig 5). Median prediction correlation of the 1Dx2-CNN model is significantly greater than the pop-LN model (signed-rank test, p value at top of panel). B. Comparison of model performance as measured by MI between predicted and actual time-varying activity [16], plotted as in A. Median MI is significantly greater for the 1Dx2-CNN model. C. Comparison of model performance as measured by LL of actual activity given predicted activity, plotted as in A. Median LL is significantly greater for the 1Dx2-CNN model. D. Scatter plot compares difference in prediction correlation between 1Dx2-CNN and pop-LN models against the difference in MI for each A1 neuron. There was some variability across individual neurons, but the difference was correlated across the population (Pearson’s r and p value from Student’s T-test at top of panel). The observation that the change in performance is similar across neurons for both metrics is consistent with the idea that the capture similar aspects of model performance. E. Scatter plot compares difference in prediction accuracy as measured by prediction correlation and log likelihood, plotted as in D. Again, the change in is correlation across neurons between metrics. (TIF) [file pcbi.1011110.s002.tif]
